# Hydrothermal transformation of SnSe crystal to Se nanorods in oxalic acid solution and the outstanding thermoelectric power factor of Se/SnSe composite

**DOI:** 10.1038/s41598-017-18508-2

**Published:** 2017-12-22

**Authors:** Hyun Ju, Dabin Park, Jooheon Kim

**Affiliations:** School of Chemical Engineering & Materials Science, Chung-Ang University, Seoul, 06974 Republic of Korea

## Abstract

The present work demonstrates the synthesis of one-dimensional (1D) Se nanorods with ~50 nm diameter by hydrothermal transformation of SnSe crystals in oxalic acid solution and suggests the reaction mechanism for this chemical transformation. SnSe particles react with oxalic acid to generate numerous Se nuclei, which crystallize into Se nanorods due to the intrinsic character of the 1D growth of Se. The resulting Se/SnSe composite exhibits outstanding thermoelectric power factor without the aid of any rare dopants, which is higher than both undoped polycrystalline SnSe and SnSe doped with Pb and Cu.

## Introduction

Se is a versatile element in the chalcogenide group that has been widely studied in various fields including chemistry, medicine, ceramics, electronics, and metallurgy. Recent advances in nanotechnology enable preparation of Se nanostructures *via* various synthesis techniques, and efforts to fabricate Se-based nanostructured semiconducting devices is highly essential to develop future technology because these low-dimensional nanostructures can replace bulk materials in various applications due to their outstanding properties^1–5^. Zhang *et al*. and Liu *et al*. reported the synthesis of Se nanoparticles that are applied to high-performance Li-Se batteries^6,7^. Nanosized Se particles used as a sensor for the effective detection of materials were fabricated by Zapp *et al*. and Ahmed *et al*.^8,9^. In another study, Chang *et al*. prepared Se nanospheres combined with Au nanorods for the efficient application of cancer radiochemotherapy^10^.

Se nanostructures can be prepared by various synthetic techniques like hydrothermal synthesis, photocatalytic process, electrochemical method, vapor-phase growth, template-assisted synthesis, etc.^11^. Chemical transformation is one of the strategies to achieve Se nanostructures with desired composition, dimension, and morphology. Transformations based on ion exchange^12,13^ and Kirkendall effect^14,15^ are popular strategies used by researchers to successfully obtain target materials. However, unlike these transformations, a stabilizer-depleted transformation enables the fabrication of unary nanostructures from binary compounds. Tang *et al*. fabricated variously shaped Te and Se nanocrystals with highly monodisperse sizes from cadmium telluride (CdTe) and cadmium selenide (CdSe) nanoplates using ethylenediaminetetraacetate (EDTA) and L-cysteine as stabilizing agents^16,17^. Zhang *et al*. reported the transformation of antimony telluride (Sb_2_Te_3_) nanoplates to Te nanoplates using tartaric acid with O_2_^18^. However till date, only a few attempts are made to obtain pure and unary nanostructures using stabilizer-depleted chemical transformation procedure.

This article reports a prospective strategy to synthesize unary Se nanostructures from tin selenide (SnSe) bulk crystals by chemical transformation reaction in oxalic acid solution. The formation of an intermediate complex between SnSe and oxalic acid, followed by its oxidation, yields Se nanostructures. One-dimensional (1D) growth of Se nanostructures is observed after the nucleation because of the intrinsic anisotropy of Se, resulting in the fabrication of Se nanorods (NRs). The thermoelectric properties of the product are also investigated and compared to those of other chalcogenide-based high-performance thermoelectric materials to confirm the potential use of the Se-based material in thermoelectric applications.

## Results and Discussion

**Figure 1:**
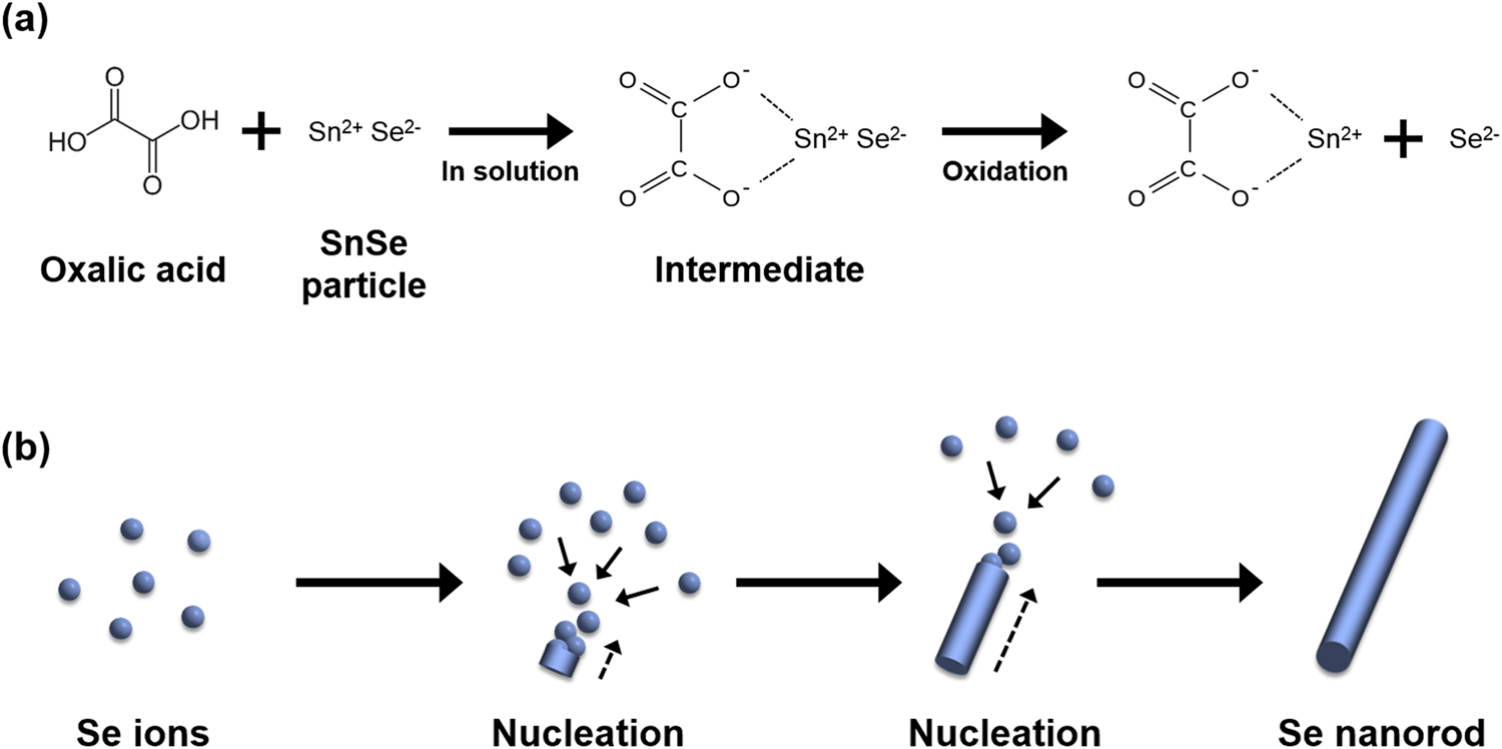
The schematic mechanism for the transformation procedure of SnSe to Se NR. (**a**) Generation of Se ions during the reaction of SnSe with oxalic acid, and (**b**) anisotropic growth of Se NR from Se ions.

The mechanism for the transformation procedure of SnSe to Se NR is schematically shown in Fig. 1. The reaction between ball-milled SnSe particles and oxalic acid in aqueous solution is triggered under hydrothermal condition, leading to the formation of a complex intermediate, as represented in equation (1).

1 $${C}_{2}{H}_{2}{O}_{4}+SnSe\to SnSe{({C}_{2}{O}_{4})}^{2-}+2\,{H}^{+}$$

This step is followed by oxidation of the metastable intermediate according to equation (2), rendering many Se nuclei in solution.

2 $$2\,SnSe{({C}_{2}{O}_{4})}^{2-}+{O}_{2}+2\,{H}_{2}O\to 2\,Sn{({C}_{2}{O}_{4})}^{2-}+2\,Se+4\,O{H}^{-}$$

Figure 1b shows the schematic for fabrication of Se NRs from Se ions through nucleation and subsequent 1D growth owing to the intrinsic nature of Se to grow anisotropic 1D structure^11^.

**Figure 2:**
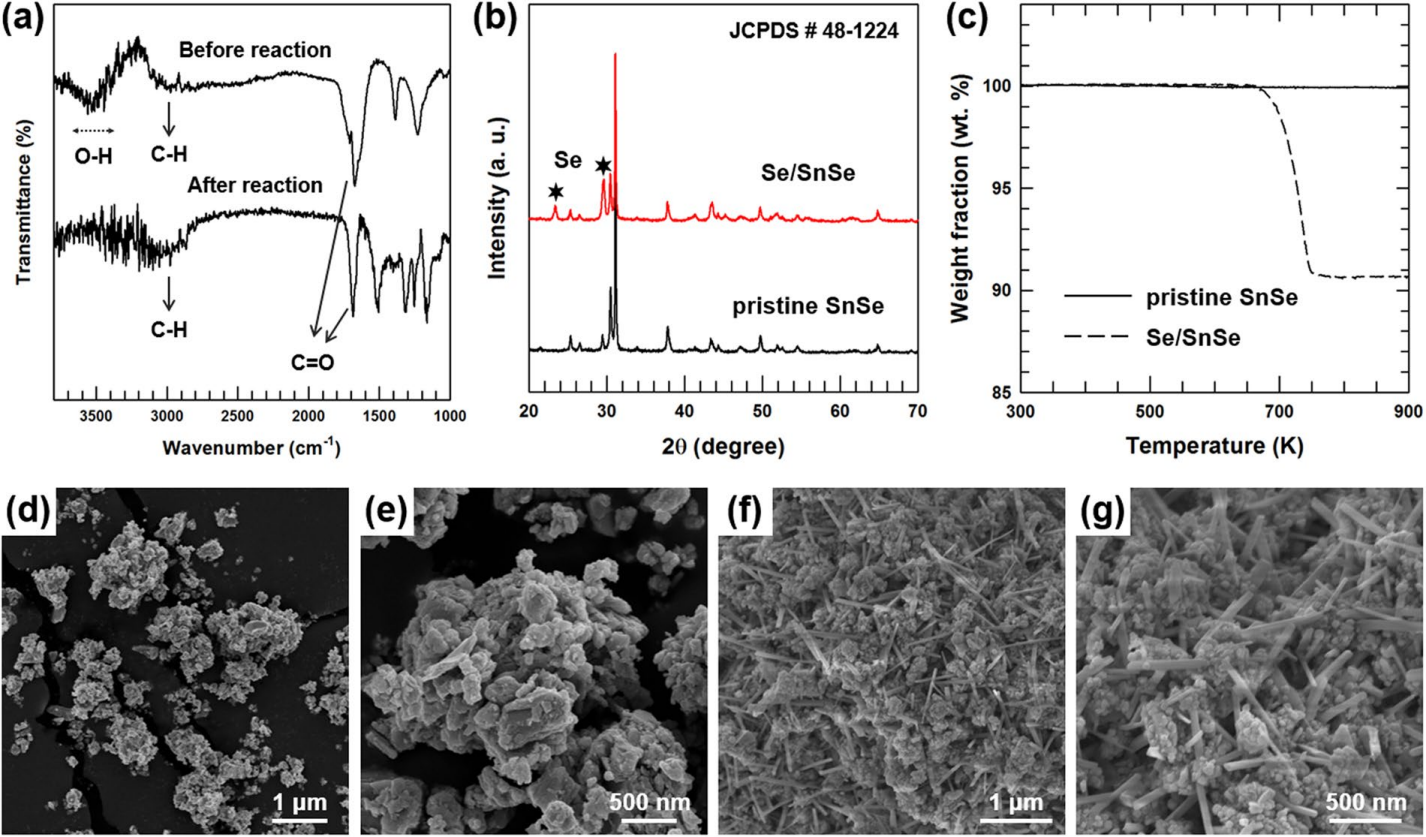
Characterization of pristine SnSe and Se/SnSe sample. (**a**) FT-IR spectra of aqueous solution of oxalic acid before and after the reaction. (**b**) XRD patterns and (**c**) TGA thermograms of the pristine SnSe powder and Se/SnSe sample. (**d**) Low- and (**e**) high-magnification FE-SEM images of SnSe powder. (**f**) Low- and (**g**) high-magnification FE-SEM images of Se/SnSe sample.

Fourier-transform infrared (FT-IR) spectroscopic analysis was performed to characterize the structure of oxalic acid in solution during the transformation to prove the proposed mechanism. Figure 2a shows the FT-IR spectra of aqueous solution before and after the reaction. A large peak at ~3400 cm^−1^ for the solution before the reaction indicates the O-H vibrations of oxalic acid^19,20^. However, the intensity of the peak at ~3400 cm^−1^ reduces after the transformation reaction because the O-H bonds of oxalic acid are broken during the course of the reaction, resulting in the formation of an intermediate complex, as represented in Fig. 1a. X-ray diffraction (XRD) and thermogravimetric analysis (TGA) results further confirm the transformation process of SnSe. Figure 2b shows the XRD patterns of the SnSe powders before (pristine SnSe) and after the reaction (Se/SnSe). Pristine SnSe powder shows diffraction patterns typical of the orthorhombic *Pnma* crystal structure (JCPDS #48-1224) with no additional XRD peak^21–23^, indicating the existence of pure phase of SnSe. In contrast, the sample collected after the reaction exhibits a second pattern of XRD peaks in addition to the primary diffraction pattern of *Pnma* crystal, originating from the pristine Se (shown in Fig. S1 in Electronic Supplementary Information), which confirms the presence of transformed Se particles in the product. Figure 2c shows the TGA thermograms for the pristine SnSe and Se/SnSe samples. The pristine SnSe, being a single component, exhibits outstanding thermal stability up to a temperature of 900 K while the Se/SnSe sample shows a thermal degradation at ~700 K, which is attributed to the transformed Se crystals. This observation is in line with the results of XRD analysis. Field-emission scanning electron microscopy (FE-SEM) images for the samples before and after the reaction further prove the chemical transformation of SnSe to Se NRs. The low- and high-magnification FE-SEM images (Fig. 2d and e) of the SnSe powder show the presence of ball-milled SnSe nanoparticles with sizes ranging from ~200 to 500 nm. Both low- and high-magnification FE-SEM images of the chemically transformed sample display the coexistence of randomly distributed nanostructures comprising SnSe nanoparticles and 1D NRs (Figs 2f,g, and S2 in Electronic Supplementary Information).

**Figure 3:**
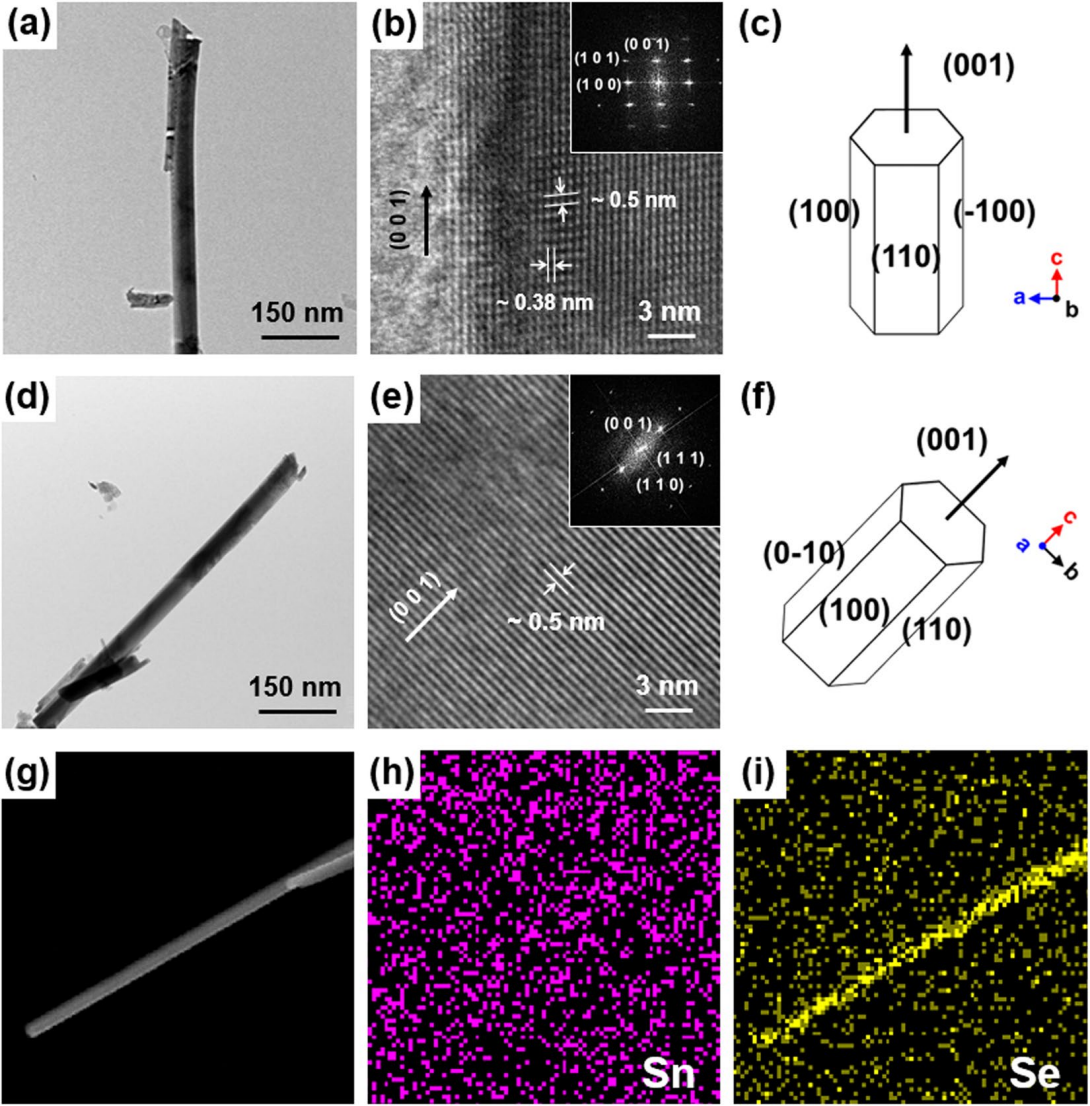
Characterization of Se NR. (**a**,**d**) Low- and (**b**,**e**) high-magnifications FE-TEM images of single Se NR. SAED patterns of the Se NRs are in the insets of high-magnification FE-TEM images (**b**) and (**e**). (**c**,**f**) Schematic illustrations of crystal structures of individual Se NRs shown in (**b**) and (**e**). (**g**) FE-SEM image of Se NRs and the corresponding EDS mappings of (**h**) Sn and (**i**) Se elements.

The NRs synthesized *via* chemical transformation were further identified by field-emission transmission electron microscopy (FE-TEM). Figures S3 and 3a and d (in Electronic Supplementary Information) show the FE-SEM and low-magnification FE-TEM images of single NR, exhibiting 1D structure with a diameter of ~50 nm. The high-magnification FE-TEM image and the corresponding selected area electron diffraction (SAED) patterns shown in Fig. 3b and e indicate that the distances between the lattice fringes are ~0.5 and ~0.38 nm, corresponding to the (0 0 1) and (1 0 0) planes of the Se NR (Fig. S1 in Electronic Supplementary Information). The direction of (0 0 1) in the SAED is parallel to the axis of NR, signifying the unidirectional growth of the NR crystal along the (0 0 1) plane, owing to the highly anisotropic structure of Se demonstrated in Fig. 1. The hexagonal crystal structures of individual Se NR can be schematically illustrated by symmetric facets represented in Fig. 3c and f; this is in agreement with the previous discussion. Furthermore, Fig. 3g–i display FE-SEM images and the corresponding energy-dispersive X-ray spectroscopy (EDS) mapping of Sn and Se atoms of Se NR, that validates the unary composition of the fabricated Se NR. The EDS spectrum of the single NR shown in Fig. S4 (in Electronic Supplementary Information) also exhibits strong characteristic peaks of Se, and only weak peaks of Sn, which is in line with earlier observations. These results confirm that the fabricated 1D NRs originating from SnSe are Se NRs and support the proposed chemical transformation mechanism described in Fig. 1.

**Figure 4:**
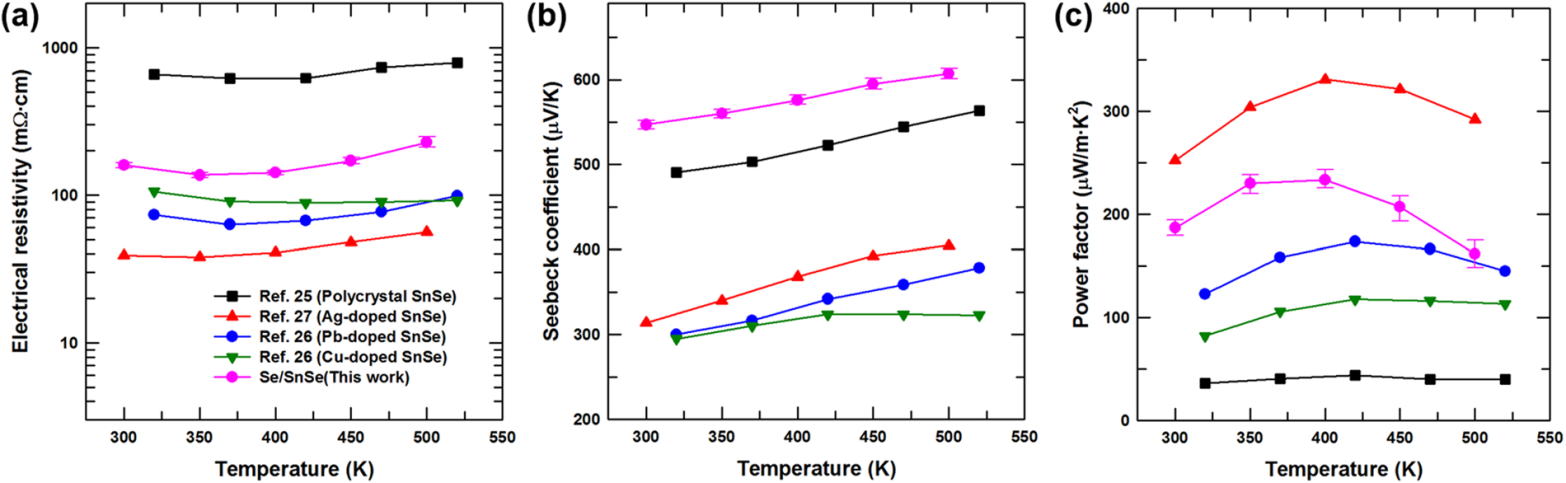
Thermoelectric transport properties of Se/SnSe sample compared to the previously reported results. (**a**) Electrical resistivity, (**b**) Seebeck coefficient, and (**c**) power factor values of Se/SnSe sample and the reported data for SnSe-based materials as a function of temperature.

The SnSe-based materials are known to exhibit outstanding thermoelectric properties^24–27^. In order to demonstrate the potential use of the fabricated Se/SnSe nanostructures as a thermoelectric material, Se/SnSe sample was pelletized to examine its thermoelectric properties, and the results were compared with those of previously reported high-performance SnSe-based thermoelectric materials. FE-SEM analysis can provide the microstructural morphology of the Se/SnSe pellet. Figure S5a shows the surface FE-SEM image of the pressed Se/SnSe pellet, revealing flat surface of the sample. High-magnification cross-sectional FE-SEM image of the Se/SnSe pellet (Fig. S5b) demonstrates that the fabricated Se nanorods are randomly distributed in the SnSe matrix, as indicated by the white arrows. Figure 4a shows the measured electrical resistivity (*ρ*) of the Se/SnSe sample and the reported data^25–27^ for SnSe-based materials as a function of temperature. The *ρ* of Se/SnSe exhibits an initial drop with increasing temperature with a subsequent increase in *ρ* value beyond the temperature of ~400 K, similar to the trends displayed by typical semiconductors. Additionally, it might be noted that the *ρ* value of Se/SnSe is lower than that of un-doped polycrystalline SnSe^25^, but higher than that of Pb, Cu, and Ag doped SnSe^26,27^. Figure 4b displays positive Seebeck coefficients (*S*) for the SnSe based materials indicating p-type semiconductor behaviors. The *S* value of Se/SnSe increases from ~550 μV/K to a maximum of ~610 μV/K at about 500 K, higher than those of the other undoped and doped SnSe-based materials. The high Seebeck coefficients in the Se/SnSe sample may be due to the potential barrier scattering of carriers at interfaces between Se and SnSe particles. Generally, the low-energy carriers cause to reduce the Seebeck coefficient, hence, their filtering at the interfaces could contribute to improve the Seebeck coefficient^28–30^. The maximum thermoelectric power factor (*S* ^2^/*ρ*) of the Se/SnSe sample is ~233 μW/m·K^2^ at 400 K (Fig. 4c), which is less than that of the optimized Ag-doped SnSe because of the relatively lower electrical resistivity of Ag-doped SnSe originating from the electrically conductive Ag atoms. However, the power factor of Se/SnSe nanomaterial is higher than both undoped polycrystalline SnSe, and SnSe doped with Pb and Cu. Measurements on five different samples prepared independently further demonstrate the experimental reproducibility of this outstanding power factor value exhibited by the fabricated Se/SnSe nanomaterial (Fig. S6 in Electronic Supplementary Information). Therefore, this work describes a hydrothermal synthesis method to transform SnSe crystals to 1D Se NRs that displays remarkable thermoelectric properties.

This study has two-fold importance as it demonstrates a fabrication procedure of 1D Se NRs from SnSe crystals *via* a chemical transformation, and emphasizes the potential use of resulting Se/SnSe nanostructure as an outstanding thermoelectric material. Se NRs were chemically transformed by treating milled SnSe crystals with oxalic acid solution. The oxalic acid reacts with the SnSe particle under a hydrothermal condition, resulting in the formation of a complex intermediate, which upon oxidation, renders Se nuclei in the solution that combine together to crystallize into Se NRs due to their intrinsic nature of 1D growth. FE-TEM images confirm that the Se NRs exhibit 1D structure with a diameter of ~50 nm. Thermoelectric properties of the Se/SnSe sample were examined and compared with those of previously reported SnSe-based thermoelectric materials. The outstanding thermoelectric power factor value of Se/SnSe sample is higher than both undoped polycrystalline SnSe, and SnSe doped with Pb and Cu, which could be attributed to the interfacial carrier scattering effect of low-energy carriers between Se and SnSe particles. This fabrication method and remarkable thermoelectric property of product can have potential application in various research areas and development of novel semiconducting devices.

## Methods

### Preparation of samples

SnSe crystals (99.999%, Alfa Aesar) were ball-milled into small particles using zirconia balls in an inert atmosphere. The rotation speed of the planetary mill was set to 150 rpm to generate a rolling action of the balls, which applied shearing forces to the materials. 29 mg of SnSe powder was added to an aqueous solution containing 180 mg of oxalic acid (C_2_H_2_O_4_) and 100 mL of DI water. After vigorous stirring, the mixture was transferred into a Teflon-lined autoclave and sealed. The vessel was then heated to 443 K for 2 h to carry out the chemical transformation. The final product was collected and washed several times with dilute HCl solution and ethanol followed by vacuum drying in the oven. The dried product was pressed for 10 min at 823 K under 50 MPa to obtain Se/SnSe samples.

### Characterization

Fourier-transform infrared (FT-IR, Bio-rad FTS-1465) spectra of the samples were obtained with an average of 32 scans in the 500–4000 cm^−1^ radiation region. X-ray diffraction (XRD, New D8-Advance/Bruker-AXS) at 40 mA and 40 kV, with Cu K*α* radiation (0.154056 nm) and a scan rate of 1°/s for 2θ ranging from 5–70°, was used to characterize the crystal structure of the materials. Thermogravimetric analysis (TGA, TGA-2050, TA Instruments) was used to investigate the thermal degradation of the samples that were heated at a rate of 10 K·min^−1^ under N_2_ atmosphere. The morphology of the materials was characterized by field-emission scanning electron microscopy (FE-SEM, SIGMA) and field-emission transmission electron microscopy (FE-TEM, JEM-2100F). The elemental mappings of the samples were performed by energy-dispersive X-ray spectroscopy (EDS, NORAN system 7, Thermo Scientific). A four-point probe method with disk-shaped compressed pellets was used to investigate the electrical resistivity. A homemade device containing a pair of thermocouples and voltmeters was used to measure the Seebeck coefficient. Five samples of the final product were prepared for the reproducibility of experiments, and the average values were reported in the manuscript.

### Data availability

All data generated or analyzed during this study are included in this paper including Supplementary Information. Raw datasets are available from the corresponding author on reasonable request.

## Electronic supplementary material

Supplementary Information
