# Supplementary material for: Hydrothermal transformation of SnSe crystal to Se nanorods in oxalic acid solution and the outstanding thermoelectric power factor of Se/SnSe composite
Source: Sci Rep. 2017 Dec 22;7:18051. doi: 10.1038/s41598-017-18508-2 (PMC5741710; doi:10.1038/s41598-017-18508-2)
Supplement: Supplementary file 1 — Supplementary Information [file 41598_2017_18508_MOESM1_ESM.pdf]

Supplementary Information:

Hydrothermal transformation of SnSe crystal to Se nanorods in oxalic acid solution and the outstanding thermoelectric power factor of Se/SnSe composite

Hyun Ju, Dabin Park, and Jooheon Kim*

School of Chemical Engineering & Materials Science,

Chung-Ang University, Seoul 06974, Republic of Korea

*Corresponding author: jooheonkim@cau.ac.kr (J. Kim)

1. Figures

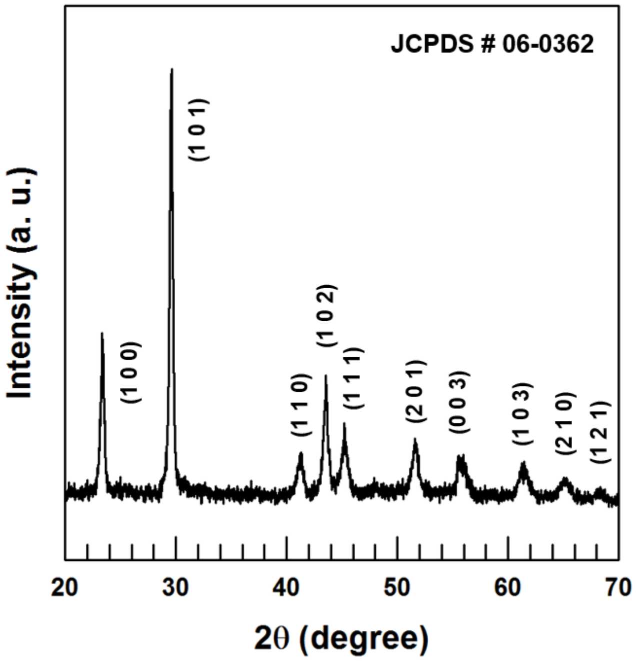

Figure S1. XRD data of pristine Se.

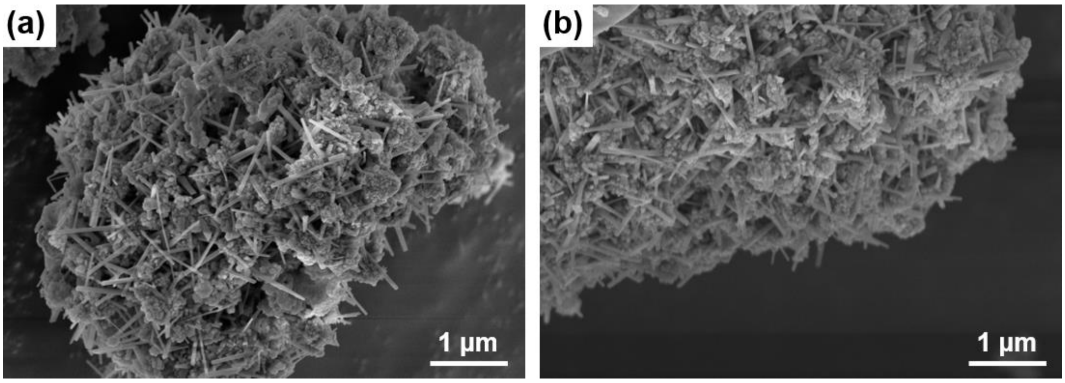

Figure S2. (a, b) Additional FE-SEM images of Se/SnSe sample. Se nanowires are randomly distributed in the sample.

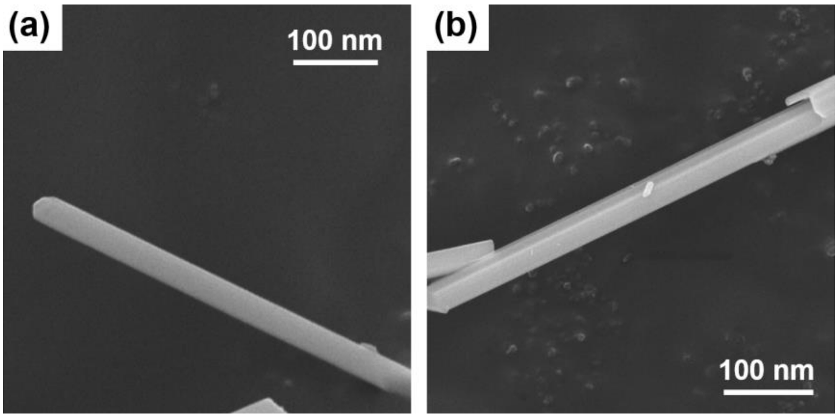

Figure S3. FE-SEM images of Se nanorods, exhibiting 1D structures with a diameters of ~50 nm.

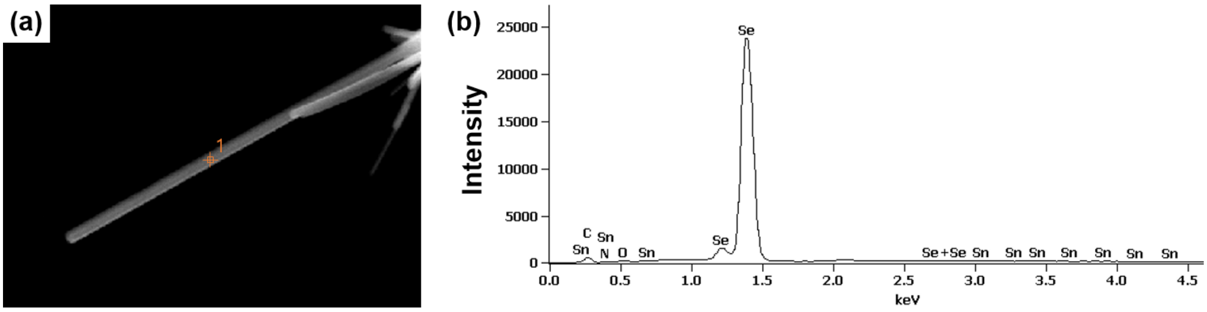

Figure S4. FE-SEM image and the corresponding EDS spectrum of single Se nanowire.

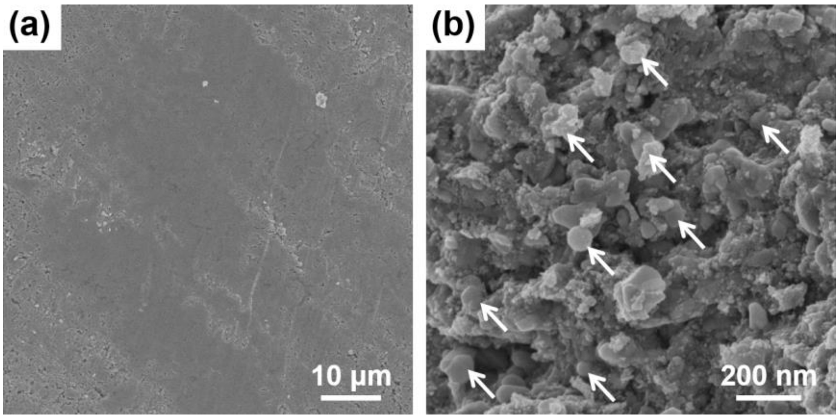

Figure S5. (a) Surface and (b) cross-sectional FE-SEM images of Se/SnSe bulk pellet. White arrows indicate the distributed Se nanorods in the SnSe matrix.

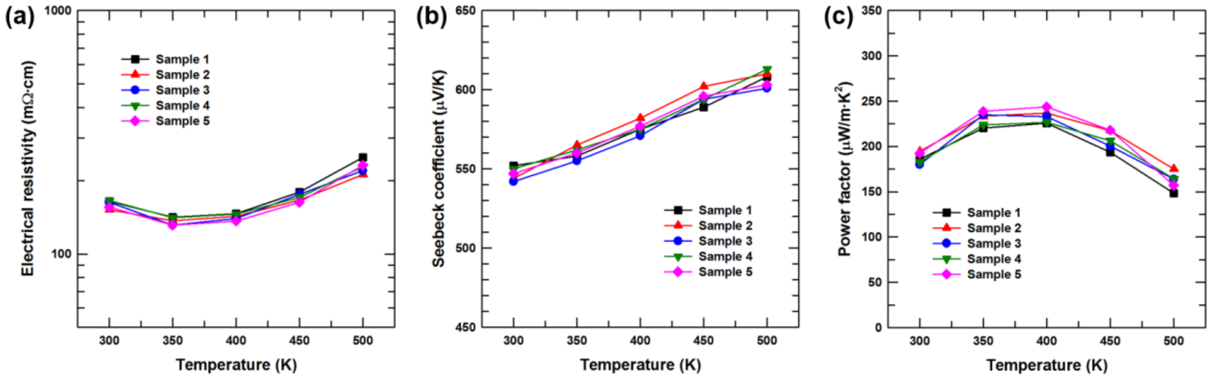

Figure S6. (a) Electrical resistivity, (b) Seebeck coefficient, and (c) power factor values of independently prepared five Se/SnSe samples as a function of temperature.
